# Comparative transcriptome-wide identification and differential expression of genes and lncRNAs in rice near-isogenic line (KW-*Bph36*-NIL) in response to BPH feeding

**DOI:** 10.3389/fpls.2022.1095602

**Published:** 2023-02-17

**Authors:** Yanxia Xue, Sajid Muhammad, Jinlian Yang, Xuan Wang, Neng Zhao, Baoxiang Qin, Yongfu Qiu, Zhimin Du, Zaid Ulhassan, Weijun Zhou, Fang Liu, Rongbai Li

**Affiliations:** ^1^ School of Electrical and Control Engineering, North University of China, Taiyuan, China; ^2^ College of Agriculture and Biotechnology, Zhejiang University, Hangzhou, China; ^3^ State Key Laboratory for Conservation and Utilization of Subtropical Agro-Bioresources, College of Agriculture, Guangxi University, Nanning, China

**Keywords:** NIL, Brown planthopper, DEGs, DELs, biotic stress

## Abstract

Brown planthopper (BPH) is the most devastating pest of rice in Asia, causing substantial yield losses and has become a challenging task to be controlled under field conditions. Although extensive measures have been taken over the past decades, which resulted in the evolution of new resistant BPH strains. Therefore, besides other possible approaches, equipping host plants with resistant genes is the most effective and environment-friendly technique for BPH control. Here, we systematically analyzed transcriptome changes in the susceptible rice variety Kangwenqingzhan (KW) and the resistant near-isogenic line (NIL) KW-*Bph36*-NIL, through RNA-seq, depicting the differential expression profiles of mRNAs and long non-coding RNAs (lncRNAs) in rice before and after BPH feeding. We observed a proportion of genes (1.48%) and (2.74%) were altered in KW and NIL, respectively, indicating different responses of rice strains against BPH feeding. Nevertheless, we characterized 384 differentially expressed long non-coding RNAs (DELs) that can be impacted by the two strains by alternatively changing the expression patterns of the respective coding genes, suggesting their certain involvement in response to BPH feeding. In BPH invasion, KW and NIL responded differently by modifying the synthesis, storage, and transformation of intracellular substances, adjusting the nutrient accumulation and utilization inside and outside the cells. In addition, NIL expressed stronger resistance by acutely up-regulating genes and other transcription factors related to stress resistance and plant immunity. Altogether, our study elaborates valuable insights into the genome-wide DEGs and DELs expression profiles of rice under BPH invasion by high throughput sequencing and further suggests that NILs can be utilized in BPH resistance breeding programs in developing high-resistance rice lines.

## Introduction

Insect pests (IPs) have always emerged as the major agricultural constraint, resulting in significant yield loss and deteriorating grain quality. Among other cereals, rice (*Oryza sativa* L.) is one of the most important crops in the Asia-pacific region and therefore, is a host to a wide range of insects that feed on it. Among these IPs, brown planthopper (BPH, *Nilaparvata lugens* Stål) is the most devastating pest of rice, accounting for about 20% to 80% of yield loss and an overall economic loss of around $300 million in Asia annually (Min et al., 2014). BPH directly causes serious damage to rice crops by sucking the sap from the conducting tissues which ultimately leads to ‘hopper burn’. Indirect damage includes the transmission of viral diseases such as grassy stunt virus and ragged stunt virus (Cabauatan et al., 2009).

So far, the effective management of BPH mainly depends on the chemical method, which is the quickest and most hard-hitting control method (Muhammad et al., 2019). However, chemical control methods are hazardous to health and our environment, which ultimately produced insecticide-resistant BPH biotypes (Tanaka et al., 2000). Hence, host-plant resistance is the most economical, effective, and eco-friendly approach to managing IPs as well as increasing crop yields (Li et al., 2019). Therefore, it is imperative to detect more novel resistant genes and then deduce the resistance mechanism. To date, 38 BPH resistance genes/QTLs have been identified in rice varieties, including African rice varieties and wild germplasm (Du et al., 2020; Muduli et al., 2021). Among them, *Bph36* is a novel BPH-resistant gene derived from two introgression lines (RBPH16 and RBPH17) developed from wild rice GX2183, which was previously reported to be resistant to BPH. Using backcrossing *via* marker-assisted selection (MAS) approach, a BPH-resistance locus on the short arm of chromosome 4 was mapped to a 38-kb interval flanked by InDel markers S13 and X48 and then was named *Bph36*. After evaluating several parameters, *Bph36* conferred high levels of antibiosis and antixenosis to BPH and confirmed that *Bph36* could be utilized in BPH-resistance breeding programs to develop high-resistant rice lines to facilitate further map-based cloning of resistant genes (Li et al., 2019).

In addition, near-isogenic lines (NILs) have been used to verify and fine-map QTLs in many crops (Kim et al., 2021) and are useful for genetic and physiological studies. NILs are predicted to be an effective means of validating a putative quantitative trait locus (QTL) by creating a NIL. Analysis of NILs that differ at QTL can be a useful tool for the detailed mapping and characterization of individual locus.

Nowadays, RNA sequencing (RNA-seq) is the most important tool to study differential expression of genes (DEGs) and long non-coding RNAs (DELs), thereby facilitating the ability to know potential physiological changes under distinct conditions. High-throughput sequencing and genome-wide alignment showed that non-coding transcripts account for the vast majority of eukaryotic genomes, most of which are long non-coding RNAs (lncRNAs) (Jarroux et al., 2017). High-throughput sequencing and computational analyses have detected novel lncRNAs in Arabidopsis (Liu et al., 2012), rice (Muhammad et al., 2019), maize (Kong et al., 2014), tomato (Cui et al., 2017), and cotton (Zhang et al., 2018), which had tissue-specificity and stress-induced expression of target genes at transcription and post transcription levels thus regulating the growth and development of organisms. Plants, being sessile, when attacked by IPs activate a defense system to resist pests through a series of regulatory mechanisms. Comparative transcriptome analysis have thus revealed the involvement of lncRNAs to regulate different defense mechanisms (Cui et al., 2017; Zhang et al., 2018).

In the present study, we conducted deep RNA sequencing to expound lncRNAs profile in rice associated with BPH feeding using the BPH-resistant NIL (KW-*Bph36*-NIL) that we previously produced (Li et al., 2019), which could further reveal the regulatory role of lncRNAs in rice response to BPH stress. We found that NIL strongly resisted to BPH feeding compared to KW. These results provide valuable resources for the study of lncRNAs in BPH stress response and will provide more insights into the biological processes of rice in the defensive mechanisms under various stresses.

## Methods

### Plant material, growth conditions, and samples collection of rice seedlings

Kangwenqingzhan (KW), an Indica rice variety developed by Guangdong Academy of Agricultural Sciences, with no resistance genes to BPH, is highly susceptible to brown planthopper (BPH) and KW-*Bph36*-NIL (NIL), a near-isogenic line, introgressed BPH-resistant gene Bph36 into KW were used as study materials. Rice seeds were germinated in a 10 cm diameter plastic cup and cultured in a greenhouse at 16/8 h day/night photoperiod under 28/25°C day/night temperature. When the rice seedlings reached 3 leaf stage, weak seedlings were removed, leaving 10 plants per cup. Then, put the transparent gauze net bag on the cup and released the 2^nd^-3^rd^ instar larvae of BPH, ensuring 8 nymphs per seedling on average. Samples were taken at 0h (control), 6h, 24h, and 48h after BPH onset, with three replicates for each treatment. Resistant materials were named as NIL0, NIL6, NIL24, and NIL48 and susceptible samples as KW0, KW6, KW24, and KW48. BPH nymphs were released at different time points, and all materials were sampled at the same time point. Samples were taken from about 5 cm of rice stem, which was eaten by BPH, and immediately put in liquid nitrogen.

### RNA extraction and illumine sequencing

Three biological replicates were used for all RNA-seq experiments sampled from each time point. Total RNA was extracted from each sample using TRIzol Reagent (Invitrogen)/RNeasy Mini Kit (Qiagen). Total RNA was quantified and qualified by Agilent 2100 Bio-analyzer (Agilent Technologies, Palo Alto, CA, USA), Nano Drop (Thermo Fisher Scientific Inc.) and 1% agarose gel. RNA with RIN value above 7 was used for further sequencing library construction. Preserved samples were sent to UniqueGene Company (Wuhan) for transcriptome sequencing. RNA-seq library was constructed by Illumina sequencing using the removed rRNA method with Ribo-Zero™GoldKits.

### Expression level estimation and differential expression analysis of genes

Rice genomic sequence (*O. sativa*_323_v7.0) and the corresponding annotations were retrieved from the Phytozome database (Version 12.0) (43). After filtering low-quality raw reads with adaptor sequences, clean reads were obtained with FASTX-Toolkit (version 0.0.14) and the purified data with high quality were mapped to the reference genome using HiSAT2 (Pertea et al., 2016). At last, the mapped reads of each sample were assembled by StringTie (version 1.3.3b) in a reference-based approach (Pertea et al., 2016). The value of fragment per kb per million reads (FPKM) was calculated to estimate the expression level of genes. For biological replicates, transcripts with |log_2_Ratio|≥1 and q<0.05 were designated differentially expressed between samples (6/24/48 h) for KW or NIL, determining up-regulated and down-regulated genes.

### LncRNA identification and target gene prediction

Based on transcript classification codes, “u” (unknown) intergenic transcripts were regarded as novel gene loci and used for lncRNAs identification. Five steps were adopted for the identification of bona fide lncRNAs as previously described (Wang et al., 2015): (1) transcripts with length ≥ 200 bp and detected in more than 3 samples; (2) transcripts derived from rRNA and tRNA were removed (cutoff E-value0.001); (3) transcripts encoding proteins and protein-coding domains were removed by the search against the Swiss-Prot and Pfam databases (cutoff E-value 0.001); (4) OrfPredictor was applied to predict ORFs and transcripts that encode more than 100 amino acids were removed (Min et al., 2005). (5) Transcripts were removed that did not pass the protein-coding-score test using the Coding-Non-Coding Index (CNCI) (Luo et al., 2014), Coding Potential Calculator (CPC) (Kong et al., 2007), and Pfam-scan analysis (Mistry et al., 2007). Transcripts without coding potential were retained as novel lncRNAs, which were used for further analysis.

The resulting lncRNA transcripts and known transcripts were then merged into non-redundant transcripts, which were further quantified by StringTie for each sample (Trapnell et al., 2012). Differential expression analysis for each sequenced library was performed using ballgown (Pertea et al., 2016). The corrected P value of 0.05 and abs |log2 (Fold change)| of 1 was set as the threshold for significant differential expression between samples (6/24/48 h) for KW or NIL, determining up-regulated and down-regulated DELs. Singular Enrichment Analysis from AgriGO was performed to identify significantly enriched GO terms in the gene list out of the background of the reference gene list (Du et al., 2010). GO term pathways with a false discovery rate (q-value) < 0.05 were considered as significantly altered. Pearson correlation was employed to explore the expression relationship between lncRNAs and their neighboring genes (≤ 10 Kb). To identify lncRNAs and putative target genes, the TAPIR tool was used with the default settings (Bonnet et al., 2010). The relationship between lncRNAs and genes was used to construct the interaction networks with Cytoscape (version 3.9.1) (Saito et al., 2012).

### Real-time quantitative PCR validation

To validate RNA-seq data, we randomly selected four genes based on their expression from DEGs and tested their expression using RT-qPCR. Total RNA used earlier for sequencing was reversely transcribed using PrimeScript™ RT reagent Kit with gDNA Eraser (Perfect Real Time) (Cat. RR047). qRT-PCR reactions were performed on a LightCycler 480 using TB Green™ Premix Ex Taq™II(Tli RNaseH Plus) (Code No. RR820). The reaction conditions were as follows: 95°C for 1 min followed by 40 cycles at 95°C for 15 s, 60°C for 15s, and 72°C for 28s. All RT-qPCR reactions were performed in triplicates. The results of each reaction were analyzed based on the 2^-ΔΔCt^ method (Livak and Schmittgen, 2001). The detection of threshold cycle for each reaction was normalized against the expression level of the rice *Actin 1* gene.

## Results

### Morphological differences of rice cultivars to BPH resistance

Brown planthopper (BPH) is the most destructive insect pest (IP) of rice, causing direct and indirect damage to the crops. Extensive measures like chemical and biological have been taken for the control of the pest during the past decades which alternatively resulted in increased BPH resistance and risk to our environment. Conversely, equipping host plants with resistant genetic resources is the most effective and environment-friendly approach to managing BPH. Therefore, the present study uses KW as a recurrent parent and the constructed near-isogenic lines KW-*Bph36*-NIL to evaluate the high-quality and complete transcriptome of rice in response to BPH. First, KW and KW-*Bph36*-NIL (referred to as NIL) were used to perform the resistance identification to BPH feeding. As shown in (Supplementary figure 1), KW was highly sensitive while NIL exhibited high resistance to BPH suggesting the involvement of NIL materials has a vital role in BPH-resistant responses. These results further illustrate the facilitation of the development of BPH-resistant rice varieties.

### Validation of gene expression by quantitative real-time PCR

In order to validate the findings of these transcripts to determine the reliability of transcriptome in gene expression, we randomly checked six genes that were influenced by BPH treatments in the two strains. Among them, three genes (Os10g0150300; Os06g0547933; Os02g0816200) were up-regulated, and three genes (Os03g0760500; Os03g0807500; Os03g0821150) were down-regulated in terms of relative expression in both KW and NIL in our RNA-seq dataset. Therefore, we further strengthen the reliability of our results by RT-qPCR analysis. Expression patterns of all the examined genes were in agreement with RNA-seq data, further indicating the credibility of our transcriptome dataset for gene exploration (**Supplementary Figure 2**). The sequences of primer are listed (**Supplementary Table 1**). The results showed that the expression trends of transcripts in both analyses were consistent indicating reliability over experimental data.

### Illumina sequencing and identification of DEGs

**Figure 1:**
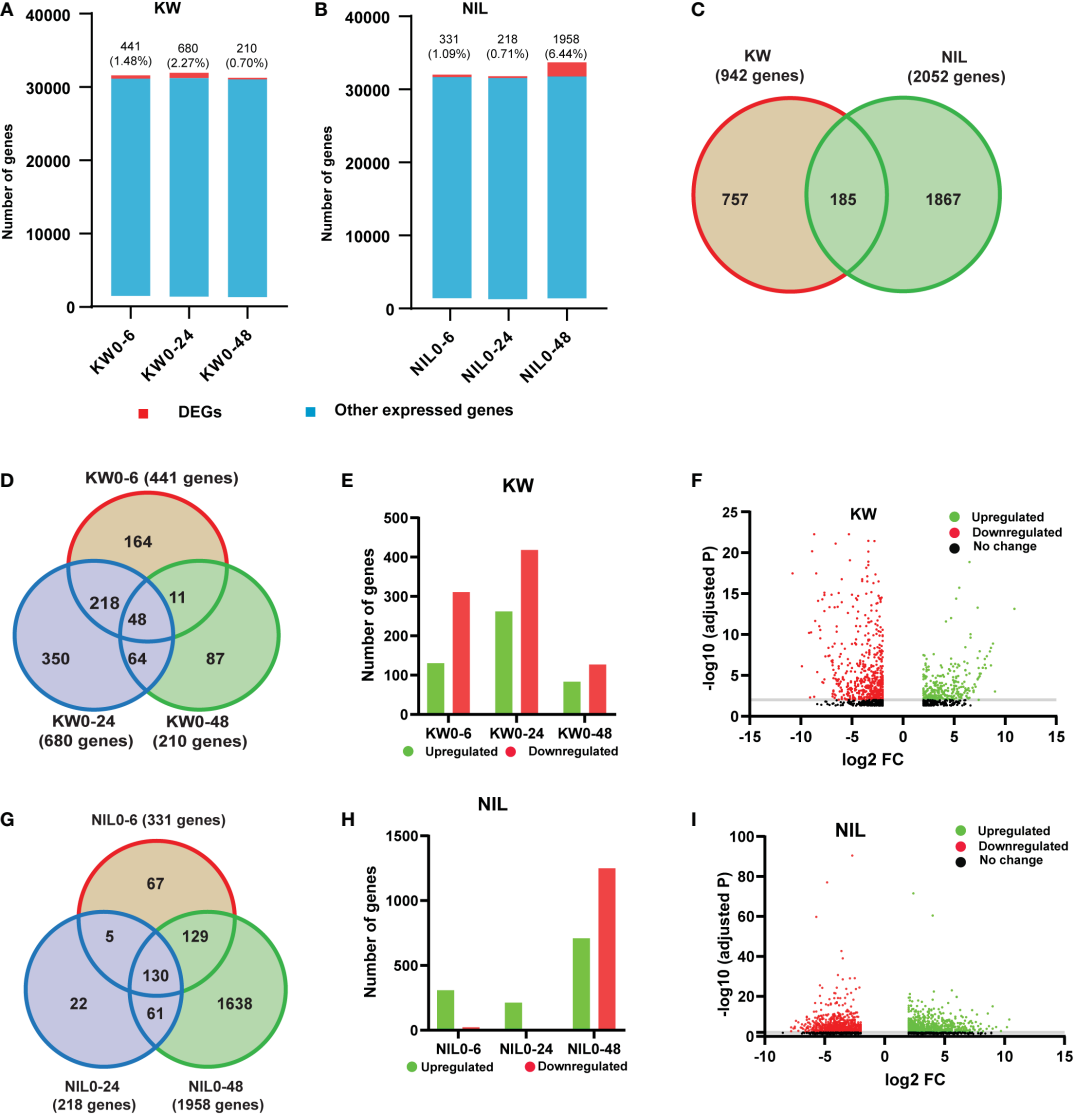
Comparison of differentially expressed genes (DEGs) in Kangwenqingzhan (KW) and its NIL under 6h, 24h, and 48h. **(A)** Proportionate percentages of DEGs to other expressed genes, red color in the bar graph shows the proportion of DEGs to other expressed genes illustrated in blue color for KW. **(B)** Proportionate percentages of DEGs to other expressed genes, red color in the bar graph shows the proportion of DEGs to other expressed genes illustrated in blue color for NIL. **(C)** Venn diagram depicts unique and overlapped DEGs among KW and NIL. **(D)** Venn diagram describing total, unique, and overlaps among DEGs after three treatments for KW, the number of shared DEGs is specified in circles. **(E)** Bars represent the distribution of up and downregulated DEGs in KW. **(F)** Volcano plot representing up and downregulated DEGs for KW. **(G)** Venn diagram describing total, unique, and overlaps among DEGs after three treatments for NIL, the number of shared DEGs is specified in circles. **(H)** Bars represent the distribution of up and downregulated DEGs in NIL. **(I)** Volcano plot representing up and downregulated DEGs for NIL.

Since we observed significant differences in the physiological performance of rice cultivars to BPH feeding, we attempted to further emphasize the alterations in the two cultivars. Therefore, we performed transcriptome high-throughput Illumina sequencing of treatments i.e., 0h, 6h, 24h, and 48h with 3 biological replicates for KW and NIL after BPH onset. In order to understand the dynamic responses of rice to BPH feeding in detail, we defined 6h, 24h, and 48h as the early stage, middle stage, and late stage of rice defense against BPH and 0h as control. Total reads and mapped reads for each replicate were estimated (**Supplementary Figure 3** and **Supplementary Table 2**). The quality and completeness of the transcriptome can have a substantial impact on annotation and downstream analyses and any miscalculation in the transcriptome assembly could affect the prediction analysis, phylogenetic signaling, and gene expression qualifications. Thus, we analyzed the quality of filtered clean reads and found that the Q30 of all samples was over 95%, indicating that the sequencing quality met the analytical conditions (**Supplementary Figure 4**). A total of 2994 DEGs were annotated in rice transcriptome after BPH feeding (**Supplementary Table 3**). Hence, it would be of great interest to find out the influence of BPH feeding on our RNA-seq dataset. Therefore, we compared the number of DEGs and other expressed genes in each cultivar against the control. The ratio of DEGs to other expressed genes was 441 (1.48%), 680 (2.27%), and 210 (0.70%) for 6h, 24h, and 48h, respectively, under BPH treatments for KW (**Figure 1A**, **Supplementary Table 4**). In comparison to KW, NIL has almost the same proportion of DEGs expressed for 6h, 331 (1.09%), and 24h, 218 (0.71%), while a surprising increase was observed for the number of DEGs at 48h treatment 1958 (6.44%) (**Figure 1B** and **Supplementary Table 5**). These results indicated that DEGs were less in number compared to non-altered genes and further implicated that the BPH feeding has impacted the GE level mostly at 48h treatment in NIL. We further ensured the specificity of DEGs between two cultivars by comparing specific and shared genes. Only a limited number of shared DEGs were identified which reflects different responses of rice cultivars toward BPH feeding (**Figure 1C**). When attacked by BPH, similar to the changes in KW, the number of DEGs increased in NIL. The total number of DEGs in NIL was higher than that in KW which indicated that the resistant and susceptible rice cultivars responded differently to the dynamic changes of BPH feeding.

We also compared the DEGs of KW at different time points to evaluate specific alterations at specific time points of the BPH feeding. We found 164 specific genes for 6h feeding, 350 for 24h, and 87 genes for 48h feeding respectively. There were also 48 genes shared between 3-time points of the BPH feeding (**Figure 1D**). Next was the assessment of all DEGs of KW into upregulated and downregulated genes to identify whether their regulation into transcription is affected or not. In this regard, we observed an exceeded number of downregulated genes than upregulated genes indicating suppression of some mechanisms involved in stress tolerance (**Figures 1E, F**). In the same context, DEGs of NIL at different time points were also compared to identify specific and shared genes. NIL exhibited 67 expressed genes for 6h, 22 genes for 24h, and 1638 genes for 48h feeding treatments (**Figure 1G**). Assessment of these DEGs into upregulated and downregulated transcripts indicated an unusual expression pattern as compared to KW treatments. The number of upregulated genes was high in the case of NIL for 6h and 24h treatments, while there was a significant increase in the number of downregulated genes observed for DEGs of 48h treatment (**Figures 1H, I**). These differences observed in the expression pattern of DEGs indicated some special responses of NIL to BPH feeding, especially at 48h treatment.

More DEGs changed dramatically in the NIL strain indicating its stronger resistance than KW. Based on these results, we may conclude that NIL activated different regulatory mechanisms compared to KW. This phenomenon showed that the two strains had different resistance patterns against BPH, NIL was mainly through positive regulation, while KW was more through negative regulation.

### Comparison of DEGs among the two strains

**Figure 2:**
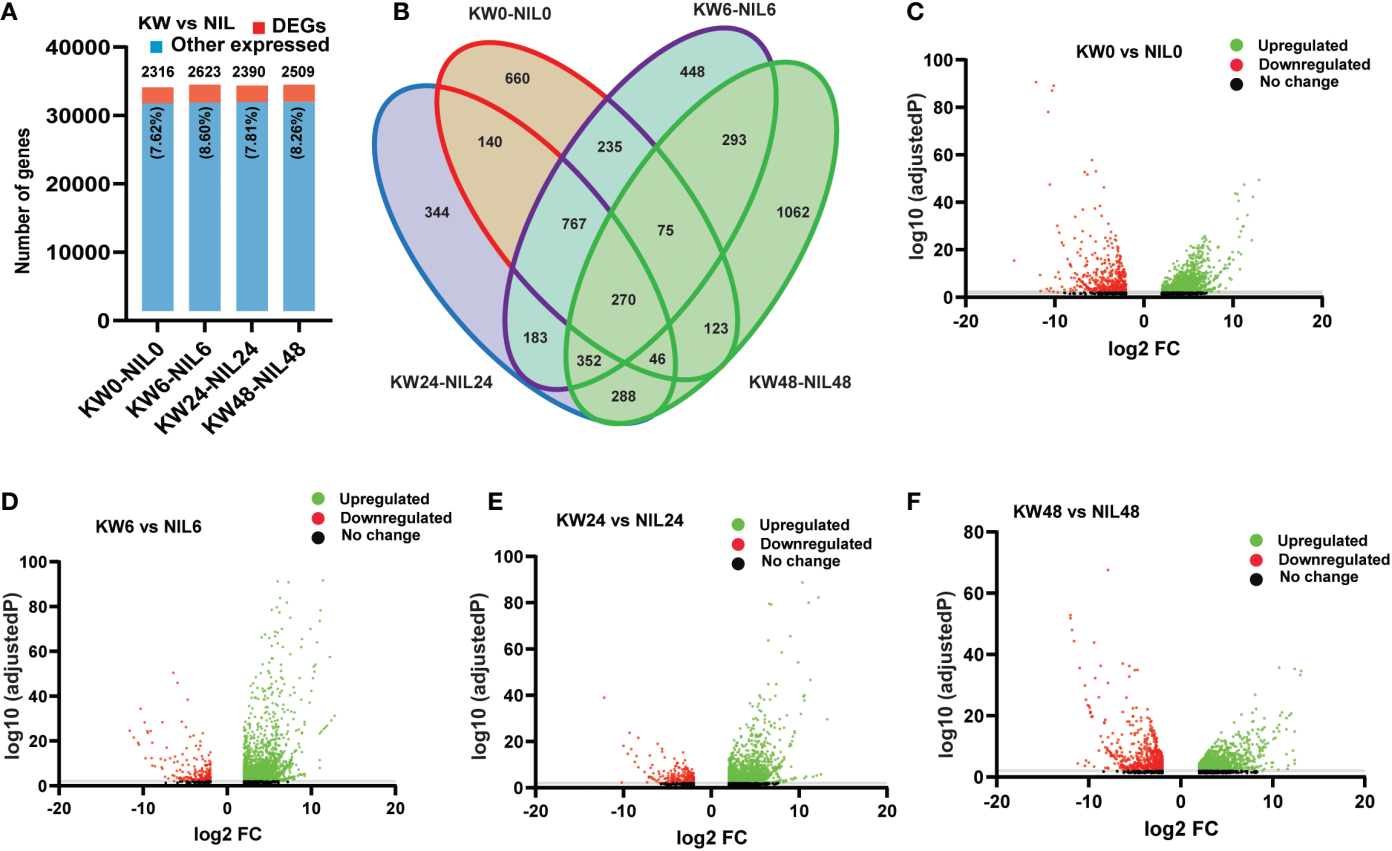
Comparison of differentially expressed genes (DEGs) among Kangwenqingzhan (KW) and its NIL under 0h, 6h, 24h, and 48h treatments after BPH feeding. **(A)** Proportionate percentages of DEGs to other expressed genes between the treatments of KW and NIL, red color in the bar graph shows the proportion of DEGs to other expressed genes illustrated in blue color. **(B)** Venn diagram depicts unique and overlapped DEGs among different treatments of KW and NIL. **(C–F)** Volcano plots representing up and downregulated DEGs for different treatments of KW and NIL at 0h, 6h, 24h, and 48h.

Hence we observed significant differences in the number of DEGs among the two strains; we wonder whether there will be differences among DEGs at different treatment times. For this purpose, we assessed combined DEGs expressed at different time points and found little differences among genes at various time points (**Figure 2A** and **Supplementary Table 6**). But, following the expression pattern of NIL for 48h, here we also observed a significant increase in the number of DEGs for 48h treatment (**Figure 2B**), which illustrates that BPH feeding has a significant impact on the number of differential genes expression at 48h. Next, we compared the regulation patterns of genes to find out up and downregulated DEGs. Among 2316 genes expressed at 0h, 1639 were upregulated, and 677 genes were downregulated. For 6h treatment, 2623 genes were expressed consisting of 2330 upregulated and 293 downregulated genes. In addition, 2390 genes were observed for 24h treatment consisting of 2065 upregulated and 325 downregulated genes. For 48h treatment, we observed a total of 2509 expressed genes consisting of 1749 upregulated and 760 downregulated genes, respectively (**Figures 2C–F**). These comparisons indicated that the differential responses were only associated with the biotype of the rice strains, however, very few changes were observed among treatments of BPH feeding.

### BPH feeding heavily impacted metabolic pathways

**Figure 3:**
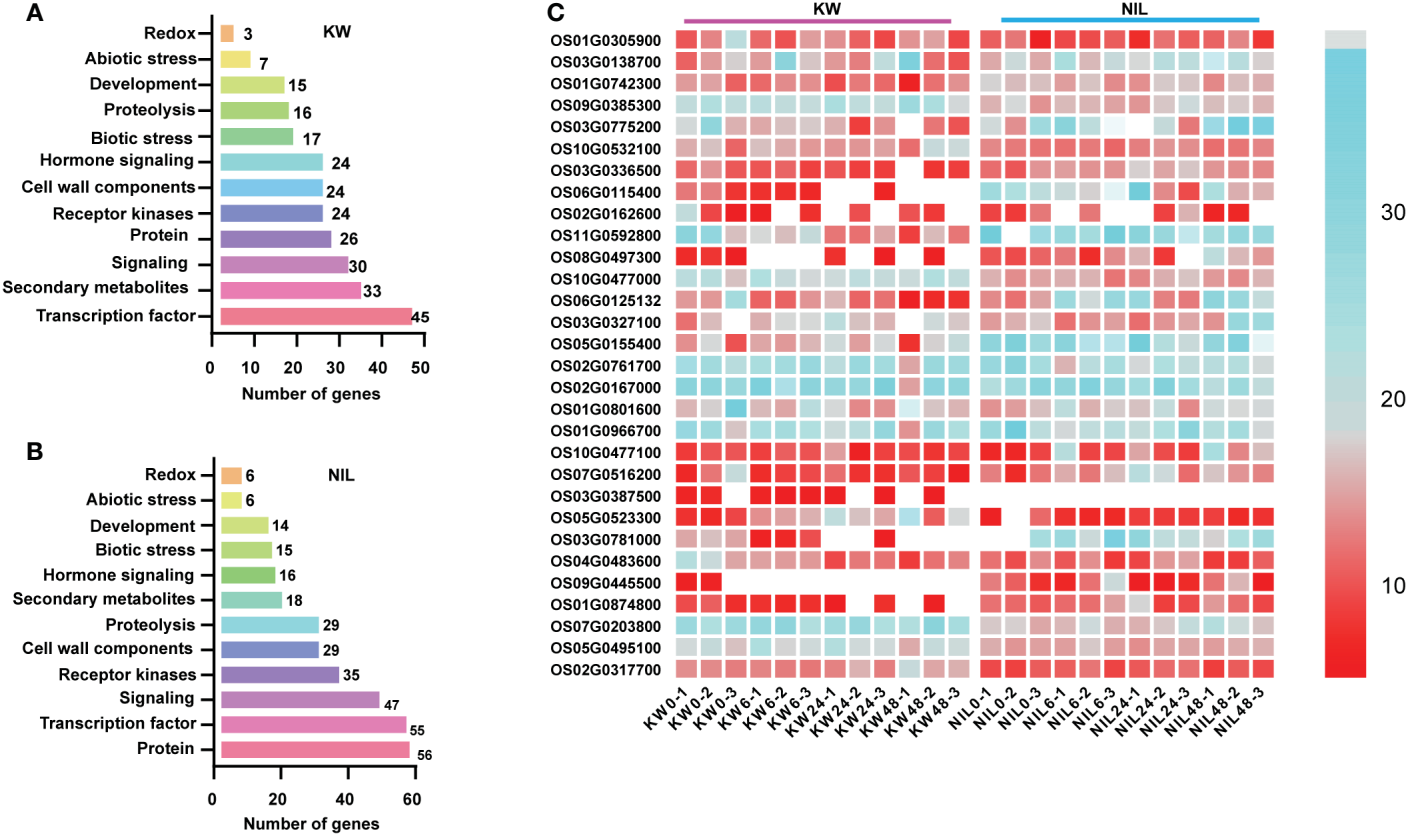
Distribution of DEGs into different pathways using MapMan. **(A, B)** Analysis of DEGs identified in different treatments of KW and NIL. The y-axis shows the distribution of genes into different pathways, while the x-axis represents the number of genes assumed for each category. **(C)** The heat map represents the expression level of DEGs identified in the study. The color scale indicates FPKM change (blue, low expression level, and red, high expression level).

In addition to be used for DEGs, RNA-seq dataset is also a good source utilized for the identification of genes involved in metabolic pathways. Therefore, we assessed DEGs involved in different metabolic pathways for two strains using MapMan software and observed the same trend as the number of DEGs with significant differences involved for different metabolic pathways. Most DEGs were observed for signaling, secondary metabolites, and transcription factor in the KW strain. However, these pathways were disturbed significantly in the NIL allowing more DEGs into it (**Figures 3A, B**).

Next, we performed hierarchical clustering of DEGs observed in KW and NIL to find out differences in the expression patterns of these DEGs. Most DEGs recorded different regulation patterns for NIL compared with KW (**Figure 3C**). Meanwhile, we observed many disease-resistant and insect resistance-related proteins and transcription factors have changed expressions, such as xylanase inhibitor protein and LRR receptor. It can also be speculated that plants began to decrease their metabolic activities while increasing their focus on coping with BPH invasion. DEGs associated with phytohormones biosynthesis about ET and SA began to multiply up-regulated expression in the middle stage, and cell wall biosynthesis showed significant changes in the late-stage suggesting that plants also initiated the relevant defense mechanism against BPH.

It was worth noting that down-regulated genes decreased sharply in NIL, probably because strong resistance to insects had developed, so previously down-regulated genes returned to normal expression levels. During the late stage of BPH onset, the DEGs of KW declined sharply while the DEGs of NIL increased significantly, forming a distinct contrast illustrating different expression patterns of the two strains against BPH feeding. These results indicated that differences in the expression pattern of DEGs are due to different strains.

### Structural comparison of lncRNAs and PCGs in two strains

**Figure 4:**
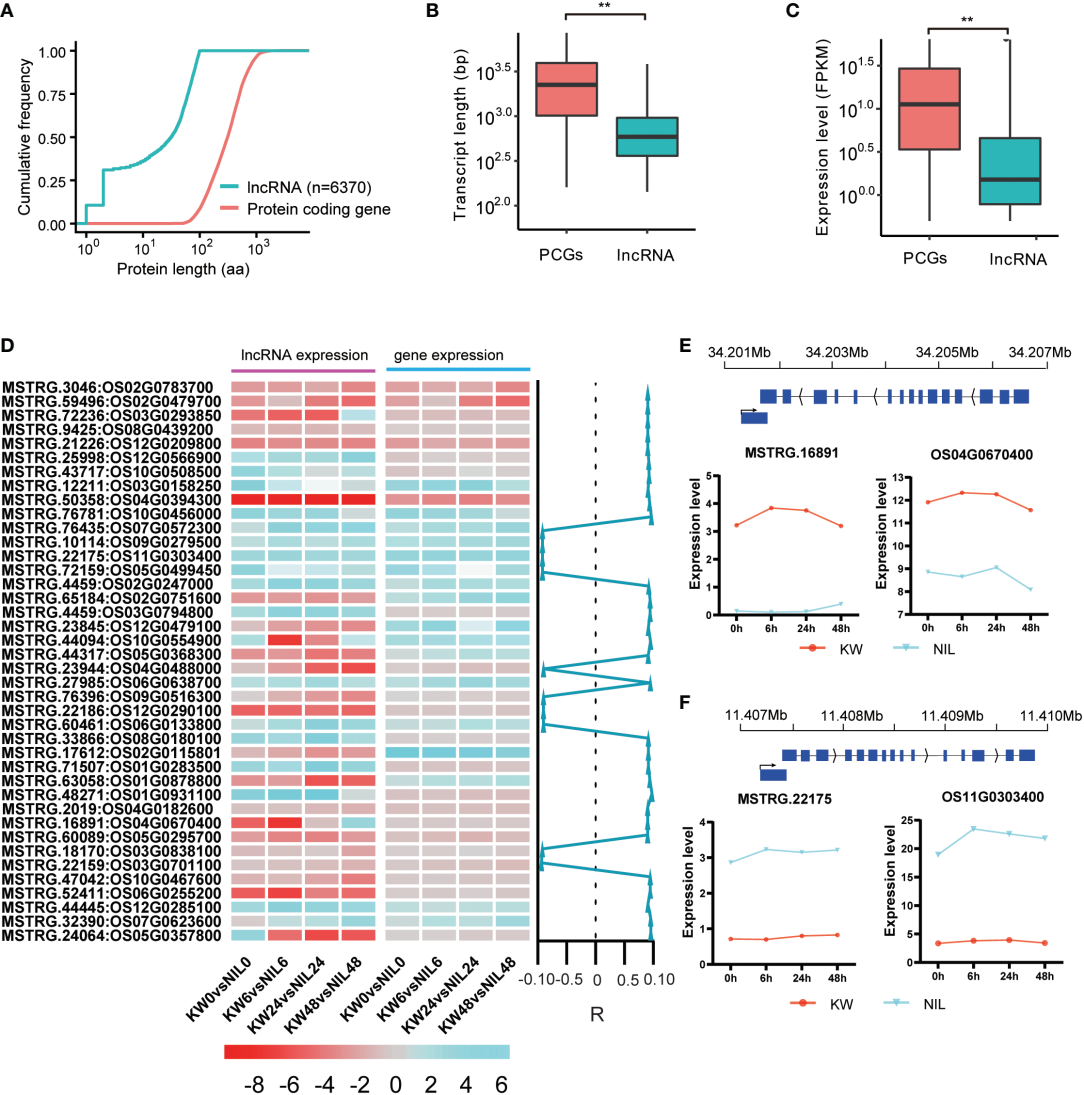
Expression profiles of differentially expressed long non-coding RNAs in rice exposed to BPH for KW and NIL. **(A)** The line graph represents the total number of predicted expressed lncRNAs and protein-coding genes (PCG). Predicted length (aa) is shown on the x-axis with scale, and cumulative frequency is revealed on the y-axis. **(B)** Predicted transcript lengths of the PCG and lncRNAs expressed in the study. ** represents the significant differences in transcript lengths between PCGs and lncRNAs. **(C)** Expression levels of the expressed PCGs and lncRNAs in the study. ** represents the significant differential expression between PCGs and lncRNAs. **(D)** Heat map represents the expression level of lncRNAs and their mediated genes in response to brown planthopper under KW and NIL treatments. Color scale indicates FPKM change (blue, low expression level, and red, high expression level). Correlation specificity score is presented on the right side of the heat map for lncRNAs and their neighboring genes. Values close to 1 mean a high correlation (R) of DELs and genes in the vicinity. **(E, F)** Predicted expression levels of two lncRNAs and their mediated targeted genes are specified. The y-axis represents expression levels of lncRNAs and PCG and the x-axis shows BPH treatments for KW and NIL.

LncRNAs have been implicated in playing a critical role in coding gene expressions (Sebastian-delaCruz et al., 2021). To predict lncRNAs in our transcriptomic dataset, we analyzed the assembled and filtered transcripts procuring approximately 6370 expressed lncRNAs in two strains after BPH feeding, with 384 differentially expressed lncRNAs (DELs) among them (**Figure 4A**). We identify these DELs including some of them related to BPH resistance in rice using PFAM, CPC, CPAT, and CNCI, which were subjected to further analysis. These DELs ranged in length between 255 and 2970 bp with the most abundant length of 300–600 bp (**Supplementary Figure 5**).

It was expected that lncRNAs and mRNAs could be evenly distributed on chromosomes, but mRNAs will have higher conservation than lncRNAs. The possible reason was that lncRNAs, as a regulatory factor *in vivo*, regulate different life activities in many ways. Further, significant differences were observed in transcript length between PCGs and lncRNAs (**Figures 4A, B**), suggesting a strong influence of lncRNAs on PCGs transcript length. We also calculated the expression level (FPKM) of the expressed PCGs and lncRNAs, depicting an average expression of 28 for the coding genes and approximately 4 for the non-coding targets (**Figure 4C**).

It has been confirmed that lncRNAs had fewer exons and were shorter in length than PCGs. The length of the expressed lncRNAs in different samples was compared with those of predicted mRNAs. An uneven length of lncRNAs was assessed in the study, however, the length distribution of mRNAs was relatively uniform (**Supplementary Figure 5**). Moreover, lncRNAs had a smaller number of exons in contrast to PCGs. The number of exons in mRNAs ranged from 1 to 14, whereas most of the lncRNAs contained only one or two exons. The number of mRNAs containing one exon was the largest (> 25000), and then decreased with the increase in the number of exons (**Supplementary Figure 6**).

### Expression profiles of differentially expressed genes and lncRNAs

Conservation of certain genes is essential for the maintenance of life activities and genetic stability. It is generally believed that mRNA is more conserved than lncRNA. Therefore, we assessed the influencing behavior of DEGs and DELs through hierarchical clustering and found that most DELs have positively regulated their neighboring coding genes (**Figure 4D**). Focusing on the comparison of KW and NIL at 0h, 6h, 2h, and 48h time points, some of the DELs, e.g., MSTRG.65184, MSTRG.44317, MSTRG.17612, etc. have a strong influence over the expression of neighboring coding targets (**Figure 4D**). Other examples of DELs that positively influenced their mediated target genes include MSTRG.16891 targeting Os04g0670400 and MSTRG.22175 mediating Os11g0303400 (**Figures 4E, F**).

**Figure 5:**
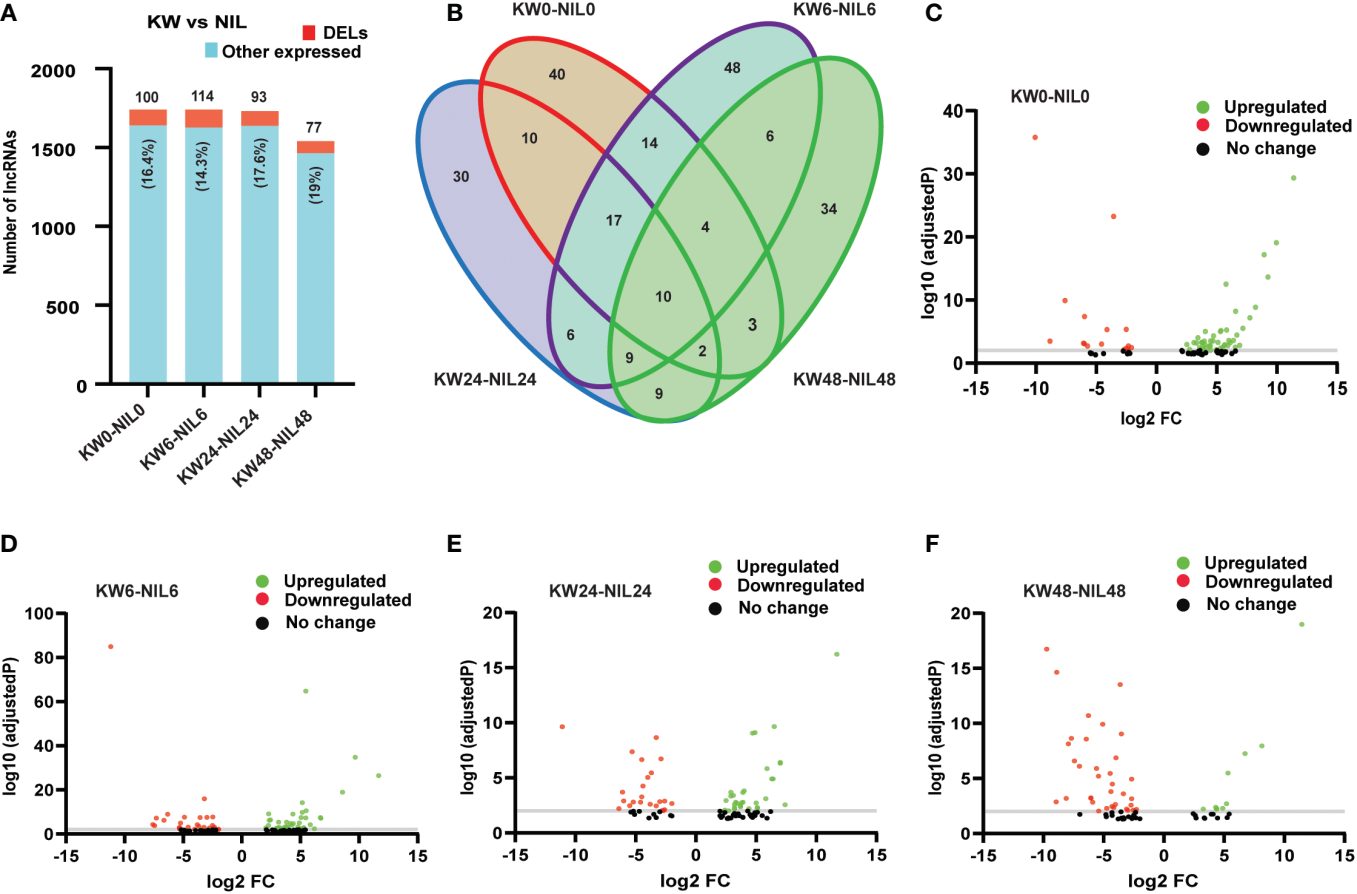
Comparison of differentially expressed long non-coding RNAs (DELs) among Kangwenqingzhan (KW) and its NIL under 0h, 6h, 24h, and 48h treatments. **(A)** Proportionate percentages of DELs to other expressed lncRNAs between the treatments of KW and NIL. Red color in the bar graph shows the proportion of DELs to other expressed lncRNAs illustrated in blue color. **(B)** Venn diagram depicts unique and overlapped DELs among different treatments of KW and NIL. **(C–F)** Volcano plots representing up and downregulated DELs for different treatments of KW and NIL at 0h, 6h, 24h, and 48h.

Hence we observed that lncRNAs have significant influences over the expression of coding genes, therefore, we wanted to check whether lncRNAs have differential expression changes between treatments of the two strains. Unlike DEGs, DELs have expressed non-significant differences among treatments, except for 48h treatment where the number of expressed lncRNAs was also less compared to other treatments (**Figures 5A, B** and **Supplementary Table 7**). These results indicated that besides differential background, lncRNAs have also changed the expression patterns of the coding targets. Comparisons of lncRNAs among different treatments showed upregulation of lncRNAs for all treatments except for 48h treatment where the number of downregulated lncRNAs exceeded upregulated lncRNAs supporting our results in the case of DEGs (**Figures 5C–F**).

### Prediction of lncRNAs targets and functional annotations

**Figure 6:**
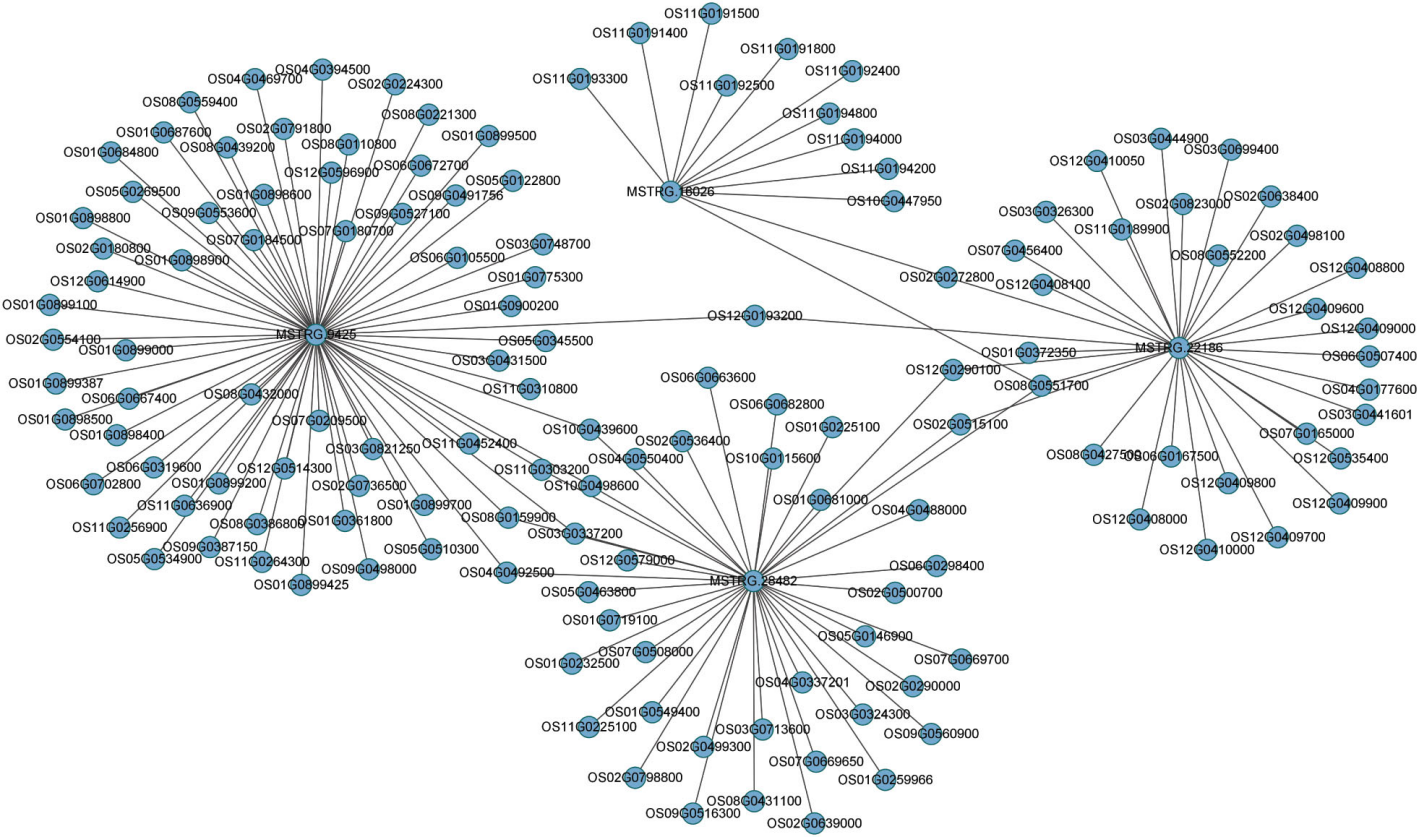
Predicted interaction network of expressed lncRNAs and PCGs. Expressed lncRNAs are depicted in the center targeting their counterpart coding genes expressed in the study after BPH treatments.

A major theme involves is the regulatory role of lncRNAs, which trigger the expression of neighboring PCGs. To predict lncRNAs and coding genes interactions, we used Cytoscape (http://www.cytoscape.org/) as visualization tool and constructed the putative interactive network of lncRNAs targeting their presumed PCGs (**Figure 6**). Correlation expression was the input data for the interaction networks between lncRNAs and their target coding genes. In the map, we presented four lncRNAs (MSTRG.9425, MSTRG.16026, MSTRG.28482, and MSTRG.22186) that targeted their interactive PCGs influencing their expression further confirmed the involvement of lncRNAs mediating coding genes targets. These analyses showed metabolic and signal transduction pathways mainly affected by DEGs, alternatively by DELs. According to previous reports, these pathways are closely related to the resistance of plants to insects, suggesting that lncRNAs can be involved in the regulation of rice resistance to BPH. These results showed that, compared to KW, NIL plants were more inclined to strengthen their defense response by expressing more genes involved in the defense process.

### Regulation of stress and signaling pathways in the two strains

**Figure 7:**
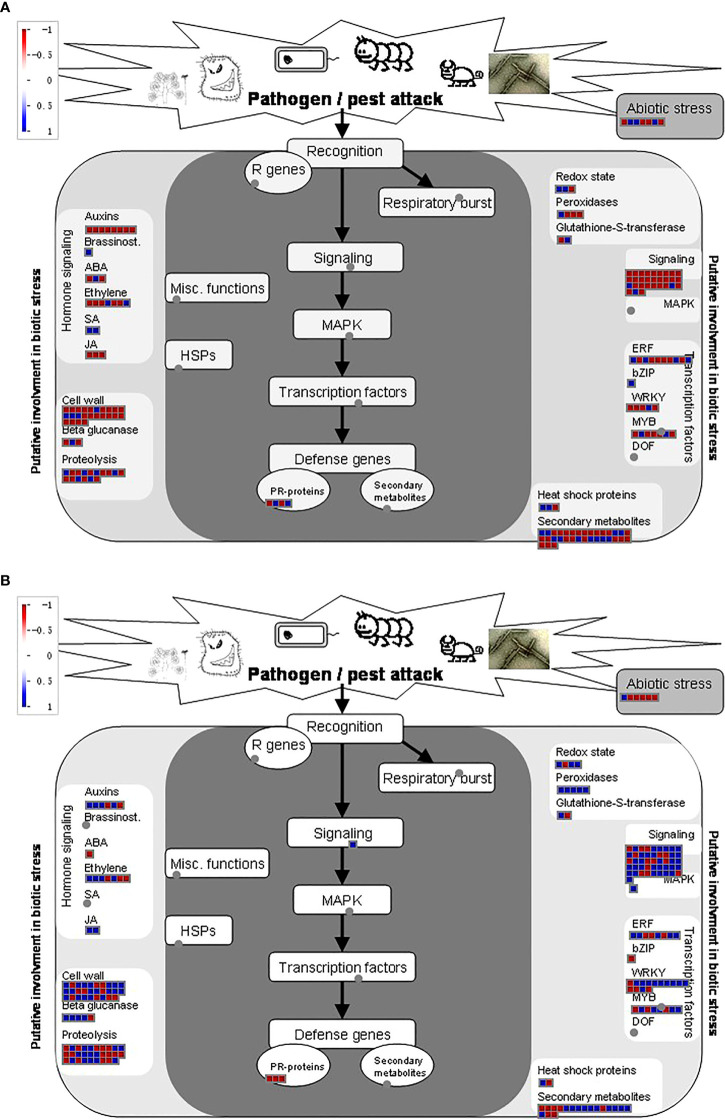
Distribution of DEGs in response to abiotic stresses among; **(A)** KW and **(B)** NIL.

Apart from other differentiation in key pathways among the two strains, biotic and abiotic stress pathways were also strongly regulated under BPH treatments. NIL has a strong influence over KW when comparing biotic stress regulatory genes. The same regulatory trend was observed for signaling genes, cell wall-related genes, and various TFs involved in our study (**Figure 7**). However, more secondary metabolites-related genes were perceived in KW compared to NIL, suggesting that pathogen attack has strongly influenced the metabolic pathways concerning the background of the strain (**Supplementary Figures 7A, B**). Moreover, BPH treatments have also impacted photosynthesis-related pathways among the two strains demonstrating differential responses of the strains toward pathogenic attack. Overall, these results demonstrate that the genetic background of rice genotypes has a strong influence over responses to pathogenic attacks and can be used as a strategy to overcome stresses.

## Discussion

Long non-coding RNAs (lncRNAs) can interact with their related protein-coding genes (PCGs) regulating their expression at functional levels (Liu et al., 2015; Deng et al., 2018; ). In rice, several studies have characterized lncRNAs and small non-coding RNAs (ncRNAs) through small RNA sequencing which regulates the expression of their related genes in growth (Chen et al., 2018), development (Liu et al., 2018), and fertility (Ding et al., 2012). However, how gene expression is controlled by DELs at the whole transcriptome level in rice against BPH feeding is still not reported. Thus, our data systemically predict lncRNAs at the whole transcriptome level for interaction in BPH stress.

Sequencing data in the current study were obtained from four individual time points (0h, 6h, 24h, and 48h) to provide more comprehensive information on the change in rice during BPH attack. High-throughput sequencing was used to analyze the changes in the response of resistant and susceptible plants to BPH feeding. Through the preliminary analysis of KW, we found that there were more down-regulated genes among DEGs and most of them were related to metabolism. On the contrary, there were more up-regulated genes as to NIL (**Figure 1**). This difference may be caused by different resistance mechanisms.

The sensitive rice had to reduce its basic metabolism levels to cope with the threat of BPH, while the resistant rice can initiate its defense mechanisms through the role of resistant genes. It is worth noting that a large number of genes were down-regulated in resistant rice at 48h after BPH onset, indicating that NIL was under great threat and could quickly adjust multiple ways to deal with the threat. Functional analysis showed that most of these DEGs were pathogen-related genes and energy metabolism-related genes (**Figure 3**).

The balance of reactive oxygen species (ROS) in plant cells needs strict regulation to ensure that cells are not damaged. In our study, several DEGs were associated with signal pathways of redox, including catalase (CAT), ascorbate peroxidase (APX), superoxide dismutase (SOD), peroxiredoxins (PrxR), and glutathione peroxidase (GPX) illustrating the main active oxygen scavenging enzymes of KW and NIL changed significantly in each stage. For example, peroxidase was up-regulated in the early, middle, and late stages of KW and significantly changed in the middle stage of NIL. Therefore, we speculate that plant can resist the invasion of BPH by decreasing the accumulation of excessive ROS in cells.

Xylanase inhibiting protein can be used as an elicitor to induce plant disease resistance and defense system. Xylanase inhibitors can help plants defense against pathogens by inhibiting the hydrolase effect of xylanases to xylan (Dang et al., 2019). At different time points of two strains of rice materials, especially in the early and middle stages of KW, there were several up-regulated DEGs related to xylanase inhibitor protein and endo-1,4-beta-xylanase A. These results demonstrated that xylanase may play a role in the response of rice to BPH invasion.

Transcription factors (TFs) are another contributor of plant resistance to stresses (Wang et al., 2012). Several differentially expressed TFs were detected in our study which has significantly impacted the transcription of adjacent genes. It has been proved that WRKY is widely involved in the transcription activation of resistance-related genes. The overexpression of OsWRKY89 could significantly improve the resistance to rice blast and white-backed planthopper (Wang et al., 2007). In our study, WRKY was significantly upregulated in the middle stage of NIL resulting in increased resistance against BPH (**Figure 3** and **Supplementary Table 8**). In our sequencing data, ethylene-responsive TFs were detected a sharp up-regulation in the early and middle stages of KW, and in the late stage of NIL, and the number of such genes in susceptible varieties was higher than that in resistant rice. These results indicated that rice activated the ethylene signaling pathway to resist the invasion of BPH. However, compared with susceptible varieties, this response mode was later and weaker in resistant rice, which may not be the main resistance mode in resistant varieties.

Phytohormones signaling pathways are another crucial stakeholder of plant resistance against stresses (Thompson and Goggin, 2006). The trend of phytohormone signaling pathways in different rice genotypes is quite different (Zarate et al., 2007; Kuśnierczyk et al., 2011; ). BPH feeding could induce a large scale of up-regulated expression of SA-related transcripts. In chickpea, hormones related to plant growth such as GA and auxin were inhibited after an insect attack (Pandey et al., 2017). We also found that genes related to SA were significantly up-regulated at the middle and late stage of KW, and gibberellin- related proteins were significantly up-regulated at the late stage of NIL, which indicated that BPH also activated the signal pathway of hormones such as salicylic acid and gibberellin. More interestingly, the expression of WRKY50 was also significantly upregulated in our study which was previously reported to regulate SA induction for systematic acquired resistance (SAR) (Hussain et al., 2018). Therefore, these hormones could also be possible participants in the response of rice to BPH.

Cytochrome P450 plays a fundamental role in plant-insect interactions in the detoxification of xenobiotics (Krishnamurthy et al., 2021). In our study, another TF, WRKY9 related to Cytochrome P450 genes changed dramatically during early and late stages in NIL and in the late stage of KW, indicating that relevant response was more rapid and durable in NIL. Some other secondary metabolites, such as flavonoids (Treutter, 2005) and isoprenoids (Vickers et al., 2009), are widely reported as signaling molecules or insect toxins to resist herbivore feeding. Genes related to the synthesis of flavonoids and isoprenoids were also enriched in the differential expression of the two strains. Genes related to cell wall metabolism were significantly up-regulated, which may be due to the fact that cell wall can provide a physical defense against piercing-sucking insect ingestion as previously reported in Arabidopsis (Zheng et al., 2013; Liu et al., 2017) and Maize (Santiago et al., 2017) (**Figure 3**).

Long non-coding RNAs (lncRNAs) are emerging as important transcriptional regulators under biotic stresses in plants (Zamora-Ballesteros et al., 2022). Here, the transcriptomic analysis revealed several lncRNAs that have responded to BPH feeding, alternatively regulating the expression of their counterpart coding genes. For example, MSTRG.65184, MSTRG.44317, MSTRG.17612, etc. are the DELs expressed in our study which significantly regulated stress-responsive genes in rice (figures 4 and 6). A strong influence of lncRNAs mediating coding gene targets in stress responses has supported previous studies (Zhang et al., 2018; Zhang et al., 2022). Therefore, the present study has provided a platform for the identification of new lncRNAs in rice under BPH attack.

## Conclusion

Altogether, this study provides a systematic analysis of DEGs and DELs in response to BPH attacks in rice. To deal with the BPH invasion as effectively as possible, plants have not only up-regulated their basic metabolism level to meet the energy demand of defense response activation but also have down-regulated their growth-related genes to balance metabolic processes. In the present study, resistant strain KW-*Bph36*-NIL had the strongest defense response after 48 hours of induction and had the maximum down-regulation of growth and metabolism-related genes following metabolic balance theory. The DEGs and DELs identified in our study can provide abundant resources for studies of BPH-resistant rice in the future.

## Data availability statement

All the raw reads produced in this study have been deposited in NCBI database with accession number PRJNA910668.

## Author contributions

RL supervised the research and designed the experiments. YX, JY, XW, NZ, and BQ helped and produced rice NILs. YX carried out phenotypic observation and other experiments. ZD and ZU performed bioinformatics analysis. SM analyzed and interpreted the sequencing data. SM, YX, and YQ drafted the article. SM, WZ, FL, and RL revised the manuscript. All authors read and approved the final manuscript.

## Supplementary material

Supplementary Figure 1 Physiological illustration of KW and NIL rice lines, **(A)** Before BPH feeding and **(B)** After BPH feeding.

Supplementary Figure 2 Validation of the expression patterns of DEGs using real-time quantitative polymerase chain reaction (RT-qPCR).

Supplementary Figure 3 Heat map represents the number of raw reads and mapped reads identified in the study.

Supplementary Figure 4 Q30 of the RNA-seq dataset.

Supplementary Figure 5 LncRNA vs mRNA length distribution in the RNA-seq dataset.

Supplementary Figure 6 Number of exons illustrated for mRNA and lncRNA in RNA-seq dataset.

Supplementary Figure 7 Distribution of DEGs in response to abiotic stresses for different metabolic pathways among**; (A)** KW and **(B)** NIL.

Supplementary Table 1 List of oligos used in the study.

Supplementary Table 2 Raw reads.

Supplementary Table 3 All DEGs identified by the RNA-seq dataset.

Supplementary Table 4 DEGs identified for KW.

Supplementary Table 5 DEGs identified for NIL.

Supplementary Table 6 Total DEGs identified among different time points.

Supplementary Table 7 Total DELs identified in the RNA-seq dataset.

Supplementary Table 8 WRKY TFs identified in the study.
