# Supplementary material for: Comparative transcriptome-wide identification and differential expression of genes and lncRNAs in rice near-isogenic line (KW-Bph36-NIL) in response to BPH feeding
Source: Front Plant Sci. 2023 Feb 17;13:1095602. doi: 10.3389/fpls.2022.1095602 (PMC9981640; doi:10.3389/fpls.2022.1095602)
Supplement: Supplementary Figure 1 — Physiological illustration of KW and NIL rice lines, (A) Before BPH feeding and (B) After BPH feeding. [file Presentation_1.pptx]

## Slide 1
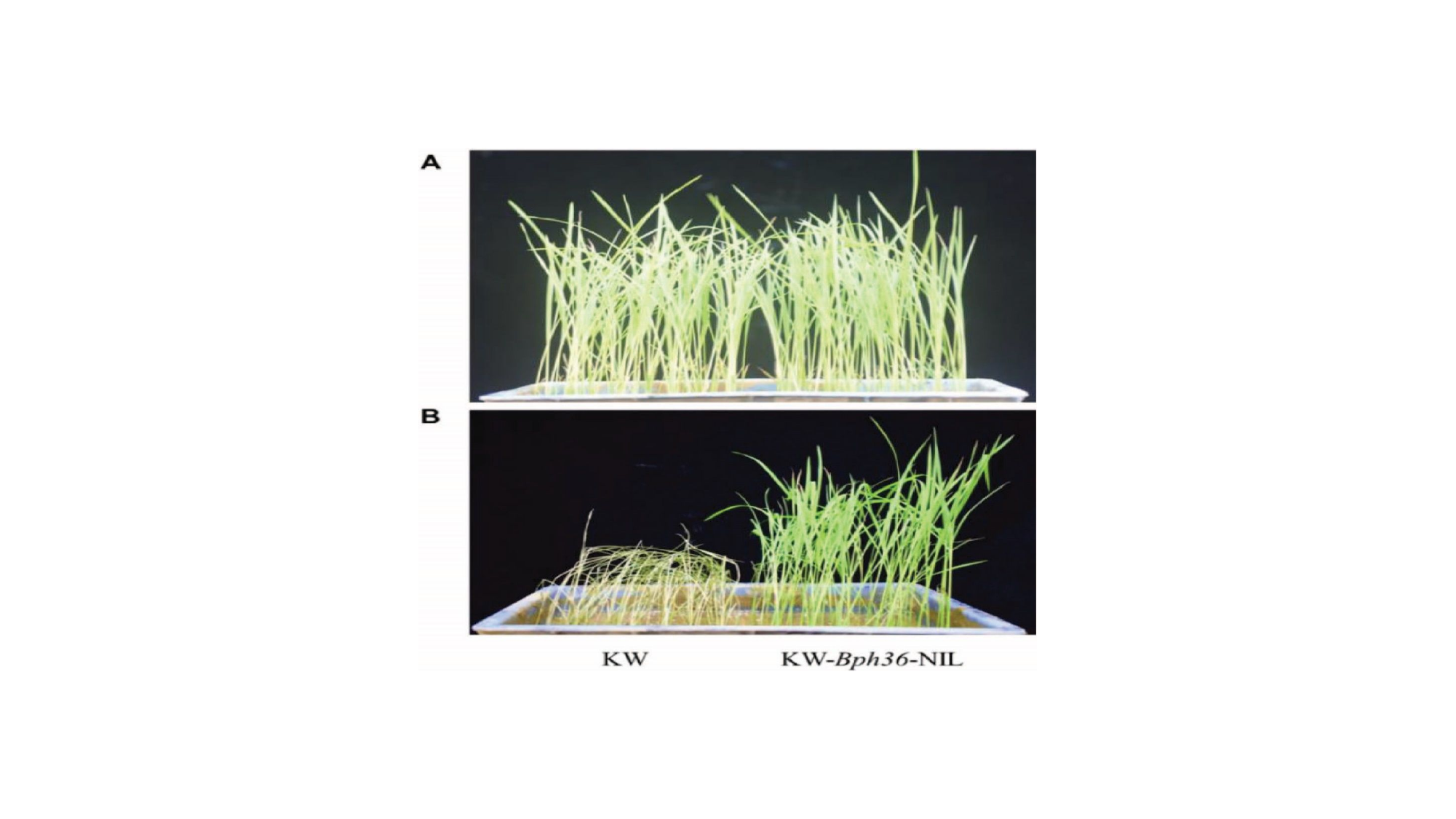

Supplementary figure 1

## Slide 2
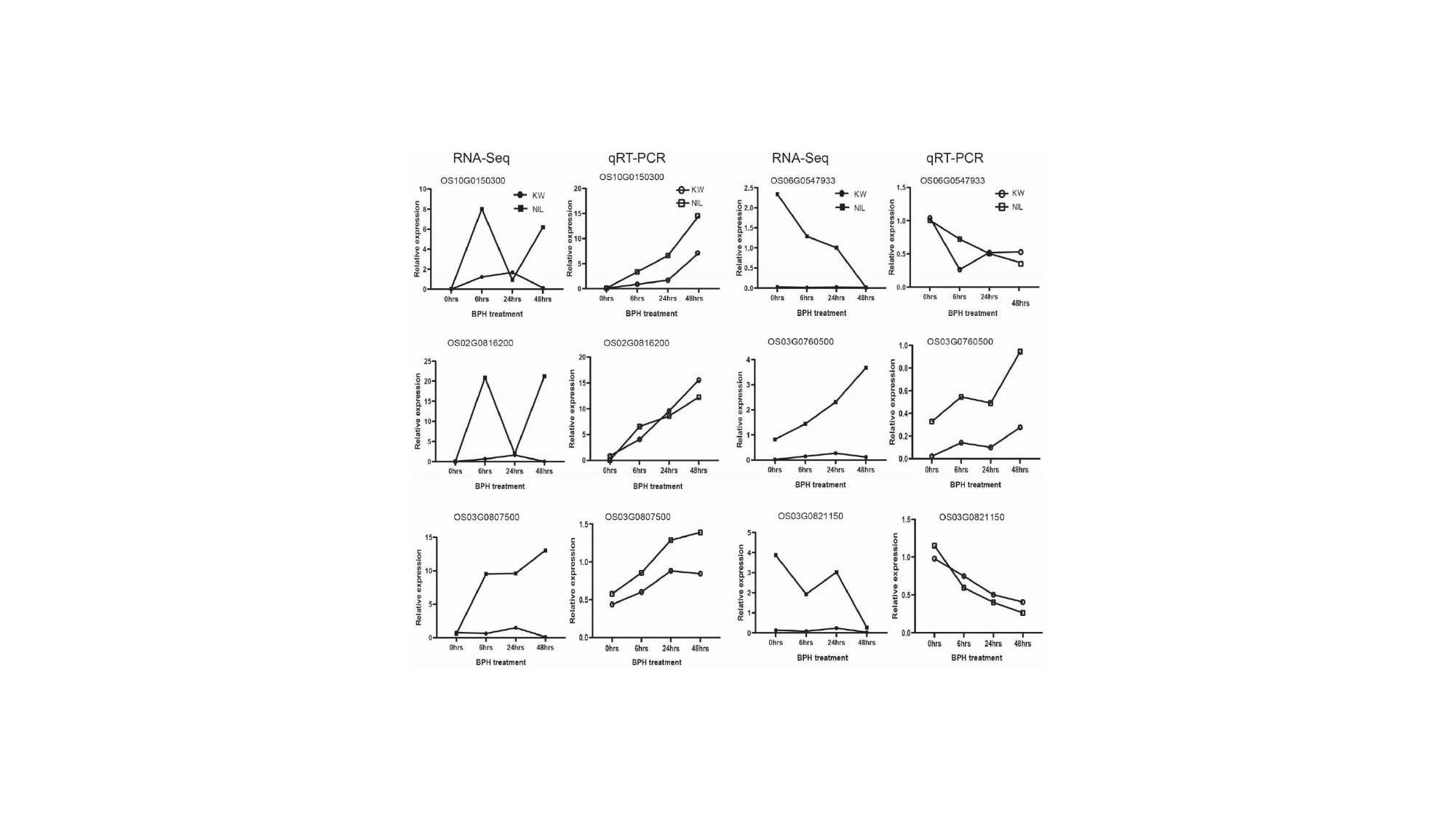

Supplementary figure 2

## Slide 3
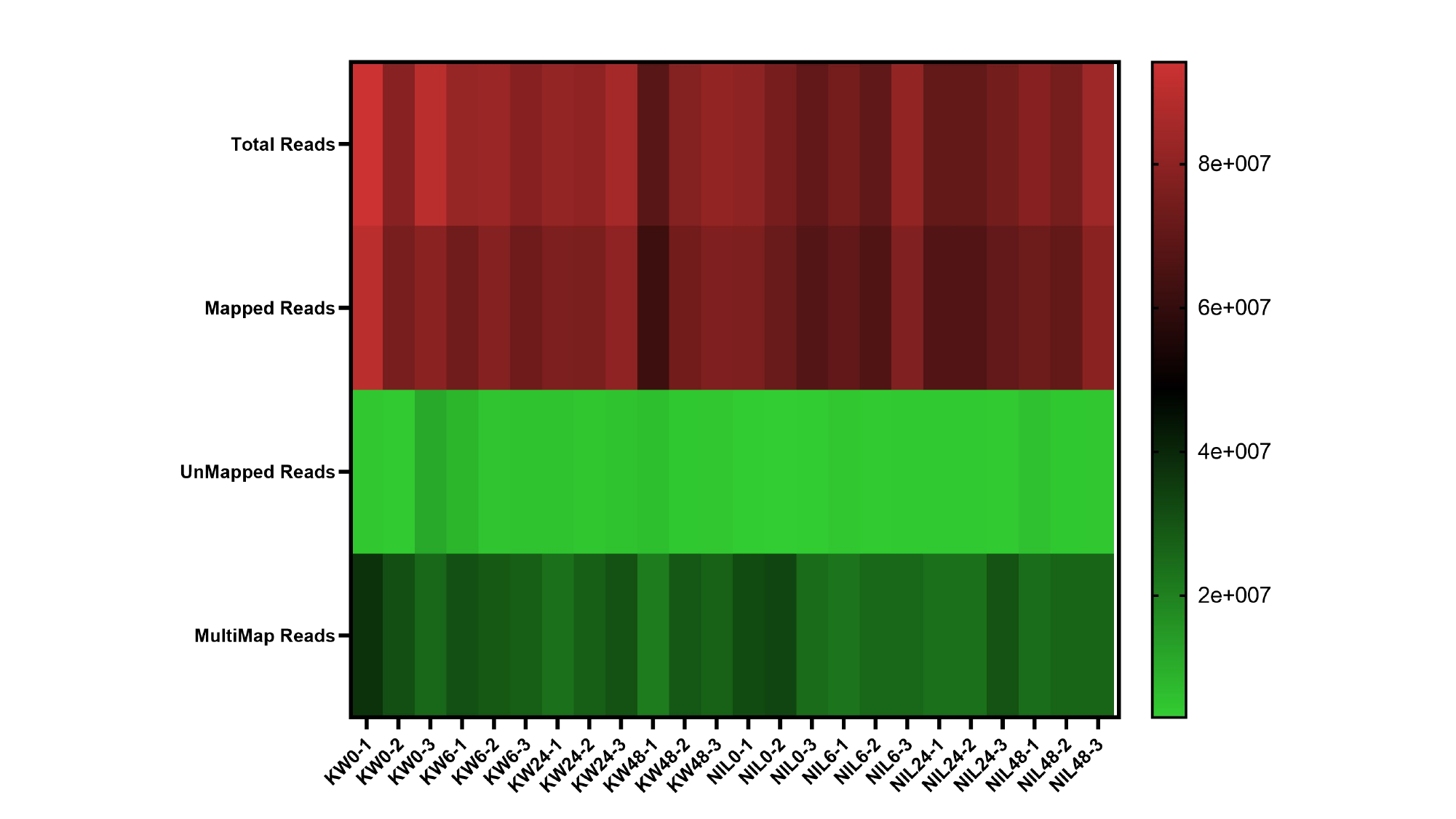

Supplementary figure 3

## Slide 4
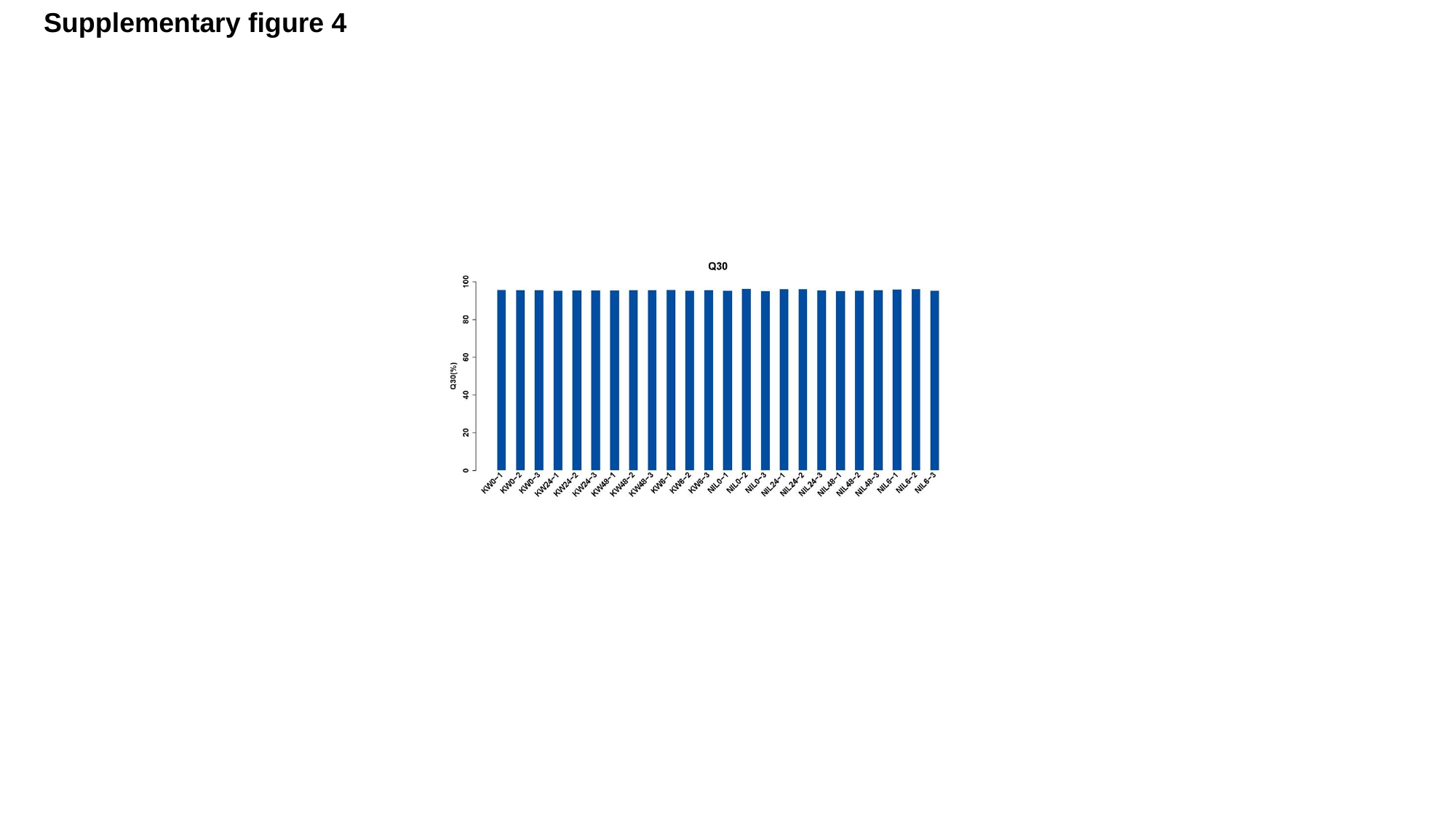

Supplementary figure 4

## Slide 5
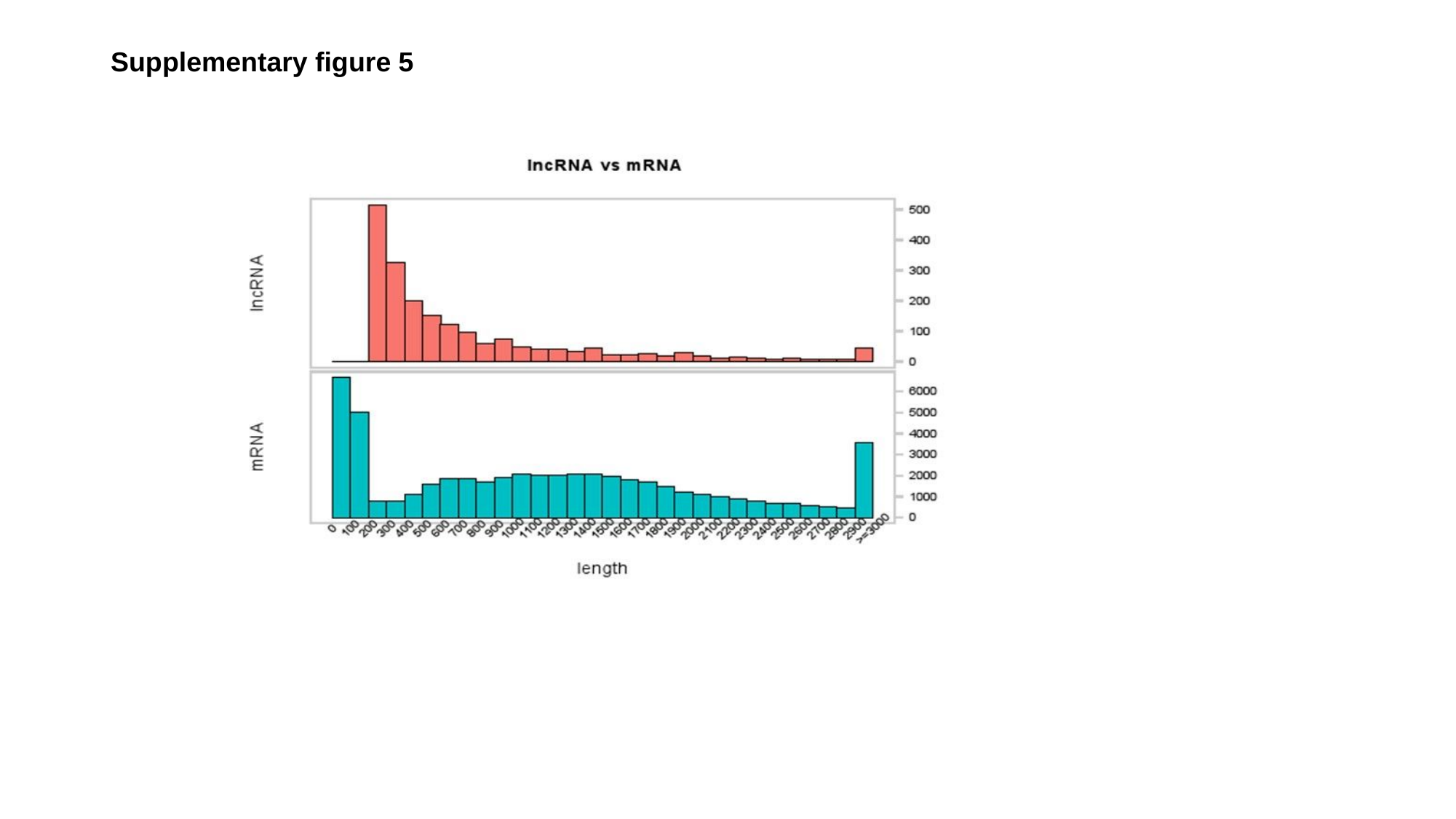

Supplementary figure 5

## Slide 6
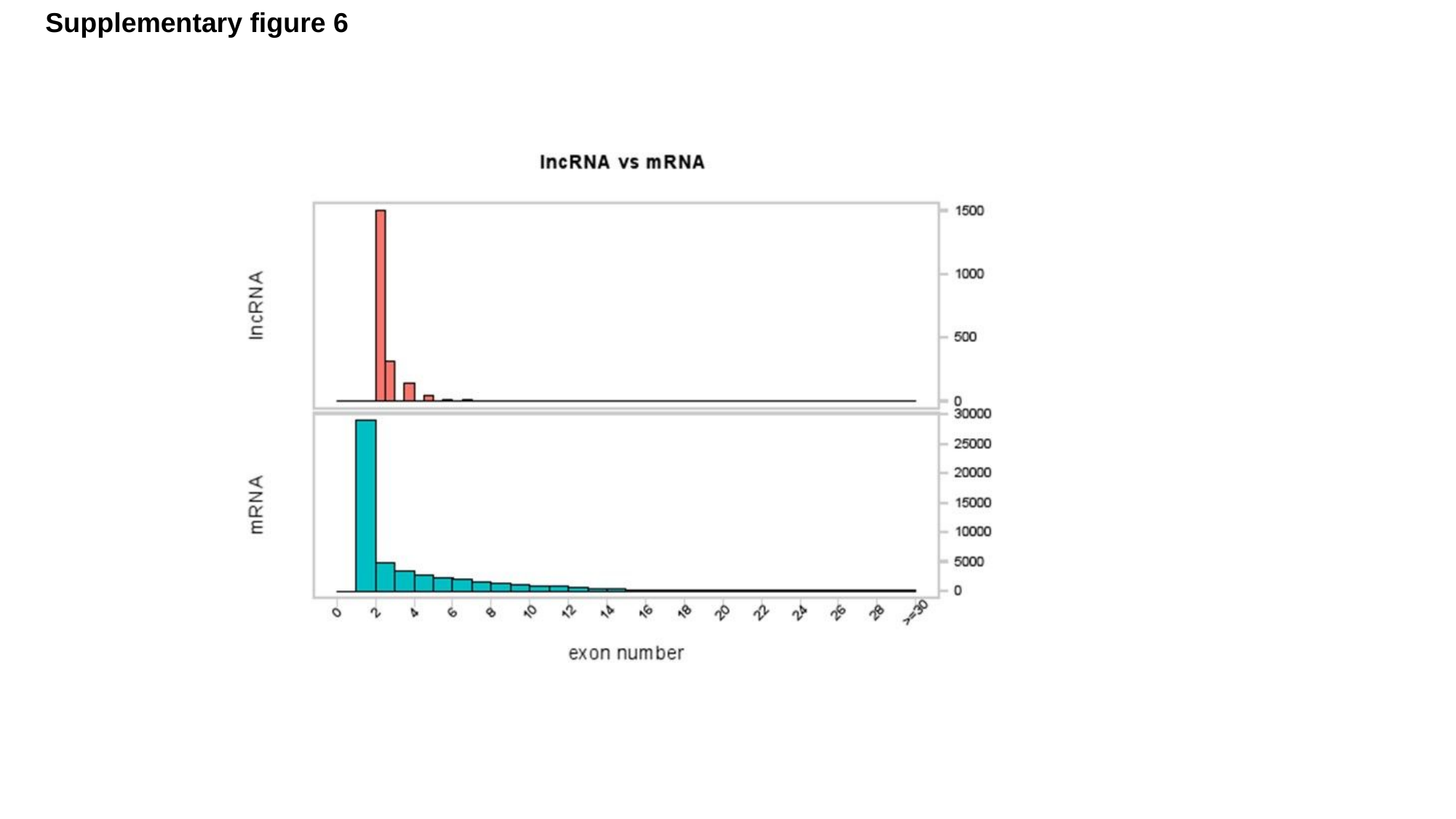

Supplementary figure 6

## Slide 7
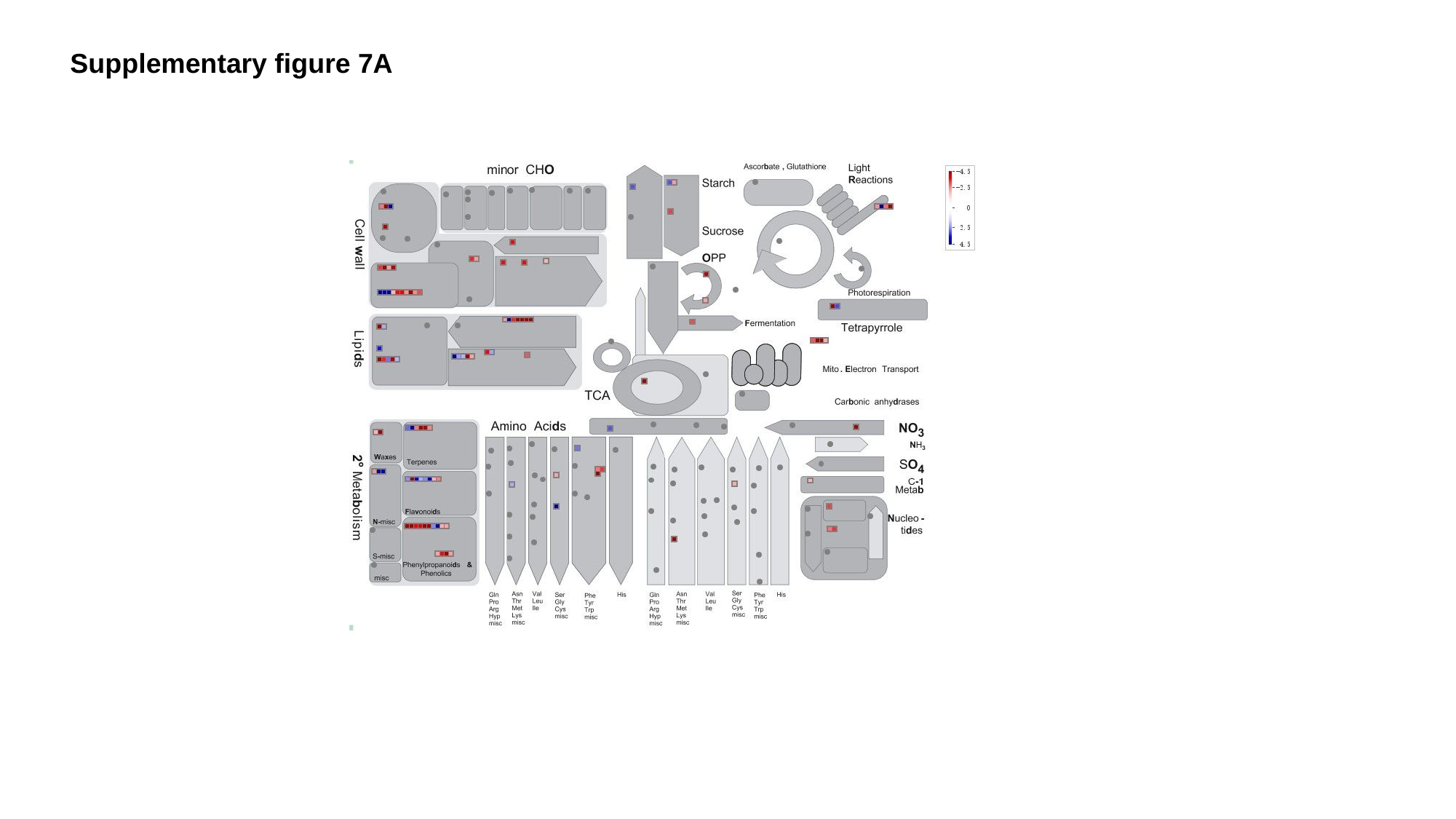

Supplementary figure 7A

## Slide 8
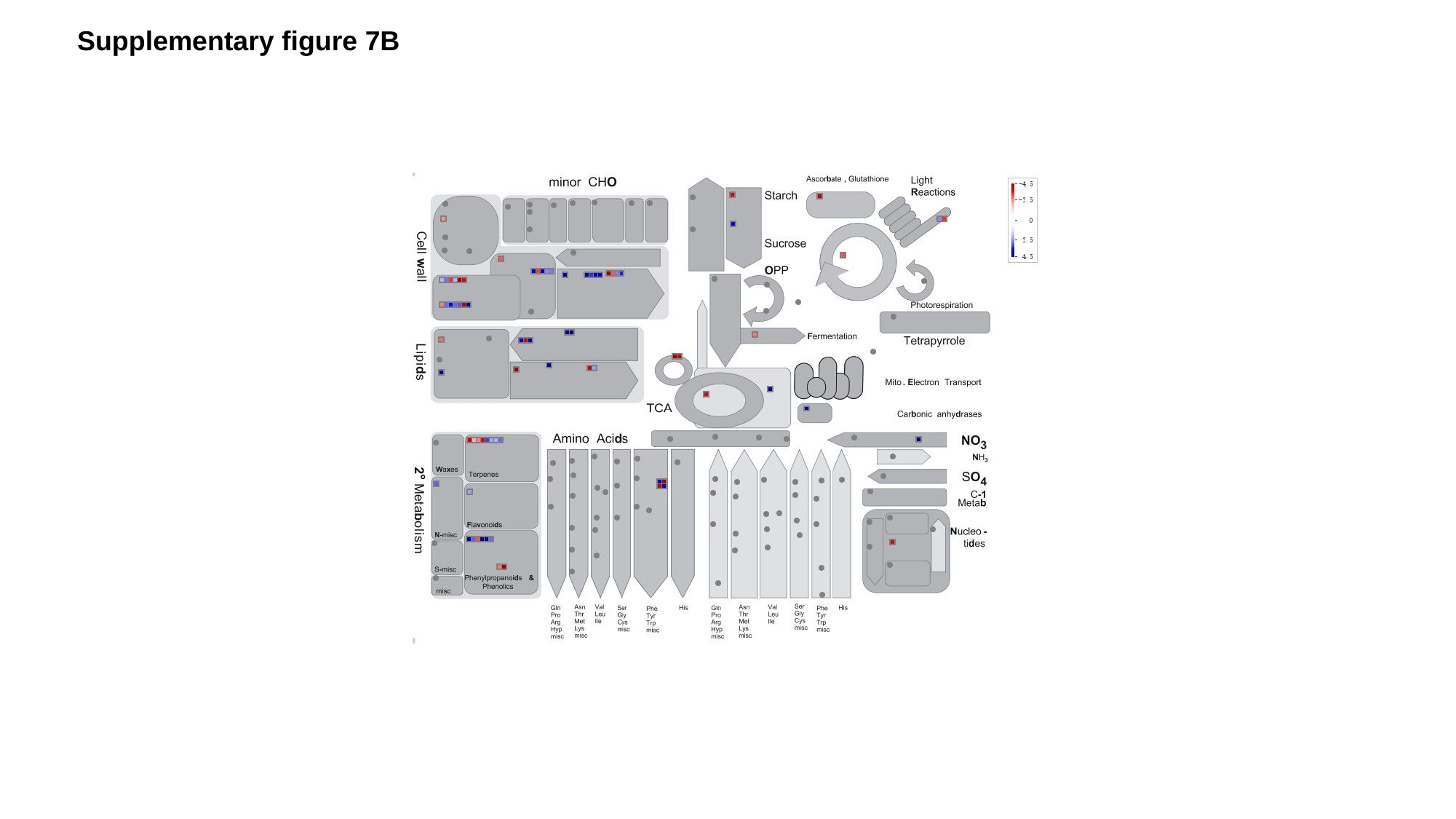

Supplementary figure 7B
